# Precopulatory but not postcopulatory male reproductive traits diverge in response to mating system manipulation in *Drosophila melanogaster*

**DOI:** 10.1002/ece3.3542

**Published:** 2017-10-30

**Authors:** Kristina U. Wensing, Mareike Koppik, Claudia Fricke

**Affiliations:** ^1^ Institute for Evolution and Biodiversity University of Muenster Muenster Germany; ^2^ Muenster Graduate School of Evolution University of Muenster Muenster Germany

**Keywords:** cost of mating, experimental evolution, sexual conflict, sexual selection, sperm competition

## Abstract

Competition between males creates potential for pre‐ and postcopulatory sexual selection and conflict. Theory predicts that males facing risk of sperm competition should evolve traits to secure their reproductive success. If those traits are costly to females, the evolution of such traits may also increase conflict between the sexes. Conversely, under the absence of sperm competition, one expectation is for selection on male competitive traits to relax thereby also relaxing sexual conflict. Experimental evolution studies are a powerful tool to test this expectation. Studies in multiple insect species have yielded mixed and partially conflicting results. In this study, we evaluated male competitive traits and male effects on female costs of mating in *Drosophila melanogaster* after replicate lines evolved for more than 50 generations either under enforced monogamy or sustained polygamy, thus manipulating the extent of intrasexual competition between males. We found that in a setting where males competed directly with a rival male for access to a female and fertilization of her ova polygamous males had superior reproductive success compared to monogamous males. When comparing reproductive success solely in double mating standard sperm competition assays, however, we found no difference in male sperm defense competitiveness between the different selection regimes. Instead, we found monogamous males to be inferior in precopulatory competition, which indicates that in our system, enforced monogamy relaxed selection on traits important in precopulatory rather than postcopulatory competition. We discuss our findings in the context of findings from previous experimental evolution studies in *Drosophila ssp*. and other invertebrate species.

## 1 INTRODUCTION

Experimental evolution studies manipulating the opportunity for sexual selection and conflict are a valuable tool to study the consequences of sexual selection and conflict on reproductive trait evolution in both males and females (Arnqvist & Rowe, 2005; Edward, Fricke, & Chapman, 2010; Kawecki et al., 2012). Pioneering the use of this approach to test predictions from sexual conflict theory, Holland and Rice (1999) showed that by eliminating the opportunity for sexual selection and conflict through enforced monogamy, males from this regime caused less harm to females resulting in higher population fitness. Also in line with sexual conflict theory, females from polygamous control populations were more resistant to male‐induced harm (Holland & Rice, 1999). Male‐induced harm in females is expected to evolve as a side effect of competition between males (Johnstone & Keller, 2000). Particularly in *Drosophila melanogaster,* receipt of male seminal fluid proteins (Sfps) causes harm in females (Chapman, Liddle, Kalb, Wolfner, & Partridge, 1995; Wigby & Chapman, 2005), but transfer is beneficial to males (Chapman, 2001; Fiumera, Dumont, & Clark, 2005, 2007; Fricke, Wigby, Hobbs, & Chapman, 2009; Fricke & Chapman, 2017). Together with the observations that Sfps show high rates of evolution (Begun, Whitley, Todd, Waldrip‐Dail, & Clark, 2000; Swanson, Clark, Waldrip‐Dail, Wolfner, & Aquadro, 2001; Swanson & Vacquier, 2002) and are key determinants of male reproductive success (reviewed in Sirot, Wong, Chapman, & Wolfner, 2015), this led to the prediction that evolution in Sfps and the postmating male reproductive traits they regulate are an underlying factor for the change in male‐induced harm observed by Holland and Rice (1999) in monogamous males. To test the prediction that male postcopulatory traits mediated by Sfps evolve in response to manipulations of the mating system, we established replicate selection lines keeping *D. melanogaster* either under enforced monogamy or polygamy. After more than 50 generations of selection, we measured a number of traits determining male reproductive success and harm inflicted on females. We expected that under enforced monogamy, males would show decreased competitive success—especially in postcopulatory traits which are strongly influenced by Sfps—and as a result also a decrease in male‐induced harm compared to polygamous males.

In contrast to males in a polygamous selection regime, males in a monogamous selection regime do not experience intrasexual competition neither on the pre‐ nor postcopulatory level. Male competitive traits are hence expected to evolve in response to the manipulation of the males’ competitive environment. Assuming that traits beneficial in intrasexual competition are costly, relaxed sexual selection on these traits is expected to result in males becoming less competitive, while males under stronger sexual selection are expected to invest in these traits increasing their competitive abilities. On the precopulatory level, males may increase competitiveness by evolving better fighting capabilities, enhance display traits or elaborate courtship behavior (reviewed in Andersson, 1994). On the postcopulatory level, traits that optimize fertilization success and paternity share in sperm competition will be selected for (reviewed in Simmons & Fitzpatrick, 2012). Such traits include testes size, number of sperm transferred, sperm morphology, sperm viability, and ejaculate allocation. Apart from sperm traits, also the nonsperm components of the ejaculate, the Sfps, play an important role in sperm competition by affecting female fecundity, remating behavior, and sperm storage (reviewed in Gillott, 2003; Poiani, 2006), and thus are expected to evolve to increase male competitiveness.

In *D. melanogaster* where females mate with multiple males (Imhof, Harr, Brem, & Schlötterer, 1998), postcopulatory competition over fertilizations is an important fitness component (Fricke, Martin, Bretman, Bussière, & Chapman, 2010), and here, together with sperm traits, male Sfps are key (Neubaum & Wolfner, 1999; Chapman, Neubaum, Wolfner, & Partridge, 2000; Fiumera et al., 2005, 2007; Fricke et al., 2009; Avila, Ram, Qazi, & Wolfner, 2010). At mating, males transfer a cocktail of >130 Sfps to the female (Findlay, Yi, MacCoss, & Swanson, 2008). Receipt of those Sfps induces a multitude of postmating behavioral and physiological changes in females (reviewed in Wolfner, 2009). Amongst other responses, transferred Sfps induce ovulation and oviposition as well as an extended refractory period in which the female is reluctant to remate. These proteins clearly confer fitness benefits to males (Chapman, 2001; Fiumera et al., 2005, 2007; Fricke et al., 2009; Fricke & Chapman, 2017) but at the same time have been shown to negatively affect female life span and reproductive success (Chapman et al., 1995), and hence, Sfps have been proposed to be mediators of sexual conflict between the sexes in *D. melanogaster* (Chapman et al., 1995; Wigby & Chapman, 2005; Fricke et al., 2009). We would therefore expect male competitive traits mediated by Sfps to evolve in response to manipulations of the opportunity for male postcopulatory sexual selection (e.g., Hosken, Garner, & Ward, 2001), but at the same time also expect male‐induced harm to evolve (Holland & Rice, 1999).

Previous studies in *D. melanogaster* mainly tested for male success in sperm competition in response to altered sexual selection regimes (Pitnick, Miller, Reagan, & Holland, 2001; Nandy, Chakraborty, Gupta, Ali, & Prasad, 2013) and traits related to sperm competition (Pitnick et al., 2001; Wigby & Chapman, 2004; Chechi, Syed, & Prasad, 2017). No response to selection in reproductive tissue investment (testes or accessory glands (AGs); the major Sfp production site) was observed in *D. melanogaster* (Wigby & Chapman, 2004), while males under sexual selection evolved larger AGs but not testes in *D. pseudoobscura* (Crudgington, Fellows, Badcock, & Snook, 2009). Using a similar experimental setup as Wigby and Chapman (2004), Nandy, Chakraborty, et al. (2013) demonstrated that males evolving under relaxed intrasexual competition were inferior in sperm competition, but again, this was not due to smaller testes or AGs (Chechi et al., 2017). Other studies testing the predictions for relaxed selection on sperm competitive phenotypes under reduced or absent sexual conflict and sexual selection using other insect species have similarly yielded mixed results with reduced sperm competitive ability in *Scathophaga stercoraria* (Hosken et al., 2001), *Onthophagus taurus* (Simmons & García‐Gonzalez, 2008), and one study in *Tribolium castaneum* (Godwin et al., 2017) but not another study in *T. castaneum* (Michalczyk et al., 2011) and *Callosobruchus maculatus* (McNamara et al., 2016). Evidence for the evolution of testes and AG size was found in *S. stercoaria* (Hosken & Ward, 2001), *O. taurus* (Simmons & García‐Gonzalez, 2008), and one study in *C. maculatus* (Gay, Hosken, Vasudev, Tregenza, & Eady, 2009) but not another study also using *C. maculatus* (McNamara et al., 2016). Hence, the question which male traits evolve due to altered sexual selection pressures is still not fully resolved. A better insight will not only help to understand the evolutionary pressures shaping male reproductive traits but might also improve our understanding of male–female coevolution.

The goal of our study was twofold: First, we wanted to understand which male traits changed in response to altered sexual selection pressures and second, whether change in male reproductive traits also changed male‐induced harm on females. We generated a high number of replicated selection lines to increase statistical power and measured a number of male pre‐ and postcopulatory traits after more than 50 generations of selection.

We strongly expected differences in male postcopulatory reproductive traits to evolve in response to our selection regimes. Contrary to our expectations, though, we did not find divergence in male postcopulatory traits between selection regimes but instead found significant changes in male precopulatory competitiveness. We discuss these results in the context of findings from other experimental evolution studies manipulating sexual conflict and sexual selection in *D. melanogaster* and other invertebrate species.

## 2 MATERIAL AND METHODS

### 2.1 Experimental evolution protocol

We established experimental evolution lines with two different selection regimes to manipulate the opportunity for sexual conflict and sexual selection: monogamy (M) and polygamy (P). We established selection lines from a wild‐type strain collected in the 1970s from flies caught in Dahomey (now Benin), Africa, which has since been kept in the laboratory at large population size with overlapping generations as a cage population. The strain was held under constant conditions at 25°C and 60% humidity with a 12/12 hrs light/dark cycle (hereafter referred to as standard conditions) on standard sugar–yeast (SY) food (100 g yeast powder, 50 g sucrose, 25 g agar–agar, 30 ml 10% Nipagin solution (100 g 4‐hydroxybenzoic acid methyl ester, 50 ml water, 950 ml 100% ethanol), 3 ml propionic acid, 1 L water).

We generated experimental evolution lines by collecting 4,200 larvae from the population cage and placing them in groups of 100 larvae per plastic vial each containing 7 ml SY food supplemented with live yeast granules. Upon adult eclosion, we immediately separated virgin females and males and assigned them randomly to selection regimes and lines. Each selection line consisted of 60 males and 60 females. In the M selection regime for each line, males and females were randomly put together as pairs and housed in individual vials (*n* = 60 vials per line), while in the P regime, males and females were combined in groups of 3♀:3♂ per vial (*n* = 20 vials per line). In total, we generated 20 selection lines, ten in each selection regime randomly numbered as M1‐M10 and P1‐P10. The 20 selection lines were maintained in two blocks, each block consisting of five M and five P lines and shifted by 1 day to make handling feasible. All lines were kept at standard conditions.

The adults within pairs or groups were left to interact freely for 4 days. On the fifth day, all females from one line were pooled—after discarding the males—to oviposit on agar grape juice plates (25 g agar–agar, 300 ml red grape juice, 21 ml 10% Nipagin solution, 550 ml water) for 24 hrs. The following day, we picked 300 larvae per line and put them at standard density in groups of 100 individuals per vial to start the next generation. Upon adult eclosion, we again collected 60 females and 60 males per line and put them in pairs or groups according to their respective selection regime, and hence, one generation took 14 days to complete. Females in the M regime were collected as virgins, while polygamous individuals were not collected as virgins. After 65 generations of continuous selection, we enforced the selection regimes only every other generation to reduce the workload of maintaining the selection lines. For generations in which the selection regime was not enforced, we transferred 500 larvae per selection line into glass bottles containing 70 ml SY food. After 13 days, we randomly collected 60 inseminated females from these bottles and set them up on agar grape juice plates to oviposit and start a new selection cycle.

Our selection regime is only effective when females in the P regime mate multiply. In order to test this, we assayed female mating frequency within our selection lines after 20 generations of selection by collecting an additional 30 males and 30 females per selection line upon adult eclosion. Adults were put together in pairs or groups according to their selection regime (*n* = 30 vials for M lines and *n* = 10 vials for P lines). Females in P regimes were marked with either a red, green, or yellow dot of acrylic paint on their thorax to be able to distinguish females in one group. Pairs and groups were left together for 4 days mimicking the adult interaction phase during the selection regime, and we counted the number of matings observed for each female by daily checking vials every 20 min in the first 7 hrs of the light phase over the entire 4 days. Pairs and groups were transferred to fresh food on the second day after the end of the 7‐hr observation phase with light CO_2_ anesthesia. We observed an average of 1.41 ± 0.06 matings for females in the M regime and 1.66 ± 0.06 matings for females in the P regime during the 4 days of adult interaction. As we did not observe flies constantly during the interaction phase, we assume that the actual number of matings per female is higher. Nonetheless, our data show that females in our selection lines did indeed mate multiply creating the opportunity for postcopulatory selection to act.

### 2.2 Tester females

We generated several inbred lines from our Dahomey population starting with a single pair and subsequent full‐sib matings for ten generations. After those ten generations, three inbred lines (Iso1, Iso2, and Iso3) were allowed to expand and maintained in glass bottles on a generation cycle of 14 days at standard conditions. We estimated the remaining heterozygosity to be ~14%. Isoline females were tested for fecundity after and reproductive behavior in a single mating to a Dahomey male against pairs of the outbred Dahomey wild type (Table 1). As Iso2 had a strongly reduced fecundity compared to the wild type, we did not include it in the behavioral tests. From the remaining two, we chose the line most similar in fecundity to the wild type (Iso3) as Iso1 and Iso3 both exhibited similar mating behavior as the wild type (Table 1). Iso3 females were used as tester females in assays throughout to assess the expression of male reproductive traits. By not testing males with females from their own selection line, we circumvented measuring the reproductive response as the outcome of the coevolved history of the two sexes, but as an expression of male competitive ability instead. Tester females provide a standardized genotype and hence allow us to quantify and directly compare the magnitude of male reproductive responses from the different selection lines.

**Table 1:** Fecundity and mating behavior of isoline females in single mating assays with Dahomey males

Cross	Fecundity (no. of eggs)	Prop. females mating	Prop. females remating	Mating latency (min)	Copulation duration (min)
Dahomey × Dahomey	111.0 ± 10.3	90.0 ± 4.7%	11.1 ± 5.2%	32.5 ± 4.2	20.3 ± 1.4
Iso1 × Dahomey	76.2 ± 7.2	95.0 ± 3.4%	13.2 ± 5.5%	22.7 ± 3.1	17.0 ± 1.3
Iso2 × Dahomey	47.2 ± 8.2	–	–	–	–
Iso3 × Dahomey	90.5 ± 13.9	85.0 ± 5.6%	26.5 ± 7.6%	18.2 ± 3.2	18.7 ± 0.7

### 2.3 Generation of experimental flies

To generate experimental flies from our selection lines for the different assays, we followed the below protocol each time to reduce maternal effects. Briefly, all lines were reared under the same standardized conditions for two generations irrespective of their selection regime. After females had oviposited on agar grape juice plates on day five of the selection protocol, we transferred them to bottles containing SY food and live yeast granules. We allowed females to oviposit for another 24 hrs and then removed them. Offspring hatching in these bottles was left to interact for 4 days without enforcing the selection regimes. On the fifth day, 60 randomly chosen inseminated females were transferred to a new bottle with SY food and live yeast. Again, the offspring from these bottles were allowed to eclose, left to interact for 4 days, and then 60 randomly chosen inseminated females were transferred to agar grape juice plates and allowed to oviposit for 24 hrs. From these plates, we collected larvae in groups of 100 per vial, and upon eclosion, males were collected and housed in single sex groups of 20 males per vial until use in subsequent experiments.

All experiments were performed in two blocks corresponding to the blocks in the selection regimes (five M and five P lines per block). We always included males from the ancestral Dahomey population in both blocks of each experiment to be able to estimate the block effect in the multivariate analyses (see below 2.4). Dahomey males were obtained by placing agar grape juice plates in the population cage for several hours. Similarly, for the generation of tester females, we set up adult males and females from the inbred line on agar grape juice plates to lay eggs for 24 hrs. The next day, larvae were picked in groups of 100 per vial, and upon eclosion, Dahomey males respective virgin tester females were collected and kept in single sex groups of 20 until the start of the experiments.

#### 2.3.1 (A) Female mating frequency and population fitness within selection lines

We assayed female mating frequency within our selection lines again in generations 51 (block II) and 52 (block I) by generating experimental flies as described above. Here, we used males and females originating from the selection lines and tested them with each other for coevolved responses. We employed a full factorial design by testing each line in both the monogamous and polygamous settings. After eclosion, adults were set up in pairs and groups of 3♀:3♂ as made in the selection regimes, with 21 females and 21 males per line and mating setting (*n* = 840 females). Pairs and groups were left together for 4 days mimicking the adult interaction phase during the selection regime, and we counted the number of observed matings for each female by checking vials every 20 min in the first 6 hrs of the light phase over the entire 4 days. The three females in the polygamous mating setting were colored with blue, pink, and green dry pigment (Sennelier No. 304, No. 604, No. 895, respectively) allowing us to distinguish between individual females. Flies were transferred to fresh food on the second day after the end of the 6‐hr observation period with light CO_2_ anesthesia.

We assayed population fitness twice: once together with the mating frequency assay in generations 51/52 (although using different females which were not colored) and once in generation 79. The experimental setup to assay population fitness was similar to the mating frequency assay. After the 4 days of observation, the females were individually transferred to fresh vials and allowed to oviposit for 24 hrs. We thereby assayed fecundity in the relevant time window matching the selection regime protocol. After 24 hrs, the females were discarded, and the vials retained for offspring to eclose and subsequently being counted.

#### 2.3.2 (B) Sperm competition experiment

This experiment tested selected males for their ability to defend their paternity (P1) in a no‐choice double mating experiment after 60 (block II) and 61 (block I) generations of selection. For each selection line, 60 5‐day‐old males were individually paired with a virgin 4‐ to 5‐day‐old tester female and observed for a mating to occur (total *n* = 1,200). Only matings that lasted at least 5 min were scored as successful. We recorded time until mating began and copulation duration for all mating pairs. Pairs that did not mate within 3 hrs were discarded. After a successful mating ended, males were immediately discarded to avoid any further matings. Forty‐eight hours later, successfully mated tester females were presented individually to one competitor male (see below) and observed for 2 hrs for a remating to occur. Again, males were discarded immediately after remating occurred. Females were left in vials to lay eggs for 4 days with one transfer to fresh vials after 48 hrs and subsequently discarded. Vials were kept for offspring to develop.

Competitor males were from a *Sb* mutant stock that carries a dominant mutation for short and blunt thoracic bristles. The *Sb* mutant had been backcrossed into the Dahomey background for four generations. By scoring adult offspring to have either a wild‐type or the *Sb* phenotype, we determined paternity shares of selected and competitor males. As the *Sb* mutation is homozygous lethal and all competitor males were therefore heterozygous for the *Sb* mutation, we had to correct offspring counts, as half of the offspring from the *Sb* father bore the wild‐type phenotype. Hence, to not overestimate the paternity share of the selected males, we corrected this by doubling the number of *Sb* offspring counted and in turn subtracting this number from the wild‐type offspring count to reflect actual paternity shares. Whenever a doubling of the *Sb* offspring counts resulted in values higher than the total offspring counts, the *Sb* values were corrected by the difference to fit the original count. The corrected values were used in all subsequent analyses.

#### 2.3.3 (C) Direct competition experiment

After 72 (block I) and 74 (block II) generations of selection, we tested the selected males’ competitive abilities when in direct competition with a *Sb* competitor male for a tester female. In contrast to the sperm competition experiment where competition is limited to the postcopulatory level, males additionally competed on the precopulatory level for access to the female. Four‐day‐old virgin tester females were added individually to vials containing one selected male and one *Sb* competitor male (both 4–5 days posteclosion). *Sb* males were colored with pink dry pigment (Sennelier No. 604) 3 days prior to the start of the experiment helping us to distinguish between the two males (we verified that coloring males with the dry pigment did not affect female mate choice prior to the experiment). We set up 30 mating triads for every selection line (total *n* = 600). Triads were kept together for 4 days, and we checked triads for matings every day after lights on for 6 hrs by doing spot checks every 15 min and recording the identity of the mating male. Based on these data, we calculated the proportion of matings gained by the selected male by dividing the number of matings observed for the selected male by the total number of matings achieved by both males in a given triad. All females for which we observed no mating and who did not produce any offspring were excluded from the analysis.

After the 6‐hr observation period on day 2, triads were transferred once to fresh food using light CO_2_ anesthesia. After 4 days, males were discarded and females transferred to fresh vials to lay eggs for 24 hrs. We kept the second set of vials from days 3 and 4 of the observation phase (hereafter referred to as vial 1) and the vials in which the females were allowed to oviposit for 24 hrs when kept singly (vial 2) and allowed offspring to develop. Adult offspring were counted from both sets of vials (1 + 2) and scored as being sired either by the selected or the competitor males based on the shape of their thoracic bristles. Correction of offspring scores were performed as described for the sperm competition experiment.

#### 2.3.4 (D) Selected males’ ability to prevent female remating

One important component of male reproductive success is the induction of a refractory period in which the female is unwilling to mate with other males. We tested selected males’ ability to delay further matings of tester females in generations 54 (block I) 55 (block II), respectively. We first mated 50–60 selected males (3–4 days posteclosion) per selection line (total *n* = 1,100) individually to 4‐day‐old virgin tester females. We recorded time until mating and copulation duration for each pair and discarded males after mating had occurred. Only matings that lasted at least 5 min were deemed successful. Pairs that failed to mate within 3 hrs were discarded. For a subset of 20 randomly chosen females per selection line, we counted the number of eggs laid within 24 hrs after a first mating to a selected male to additionally determine female fecundity induced by selected males.

Ten randomly chosen females previously mated to a male from one of the selection lines were given the opportunity to remate 24 hrs after the first mating. For the remaining females, we similarly offered remating opportunities 48, 72, and 96 hrs after the first mating by randomly choosing subsets of 10 mated females each. At each time point, we transferred females individually to new vials containing one Dahomey wild‐type male (4–5 days posteclosion). We counted successful rematings within a 90‐min observation window at each opportunity.

#### 2.3.5 (E) Induction of female harm by selected males

In generations 62 (block II) and 63 (block I), we tested for differences between selection regimes in the cost of mating inflicted by selected males on tester females. To determine costs of mating, 1‐day‐old virgin tester females were paired individually with one freshly eclosed selected male and continuously housed together until the female's natural death. We set up a total of 20 pairs per selection line. Males were replaced every week with fresh 4‐day‐old males from respective selection lines to avoid confounding effects of male age. Pairs were transferred to fresh food every other day without anesthesia. At every other transfer, we kept the vacated vials to count the number of adult offspring eclosing. For the first 5 days, though, we kept all vials as this covers the adult interaction time of the selection regime. Adult offspring counts were used as an estimate of female lifetime reproductive success (LRS). We recorded female survival by daily checking for dead females. If a male was found dead, it was replaced immediately with a new male. Females that escaped or were accidentally killed during the transfer were excluded from the analyses (*n* = 20).

#### 2.3.6 (F) Male body size

We used wing length as a proxy for body size to determine whether males from the two selection regimes differed in size. To measure wing length, we froze 20 adult males from each selection line in generation 85 and cut off their left wings at the base, placed them on a slide in phosphate‐buffered saline (Calbiochem), and photographed them at 50× magnification (Observer.Z1 with Axio Vision software release 4.8.2; Zeiss Microscopy). The length of the third longitudinal wing vein between the anterior cross‐vein and the wing margin (Gidaszewski, Baylac, & Klingenberg, 2009) was determined in pixels from images using ImageJ (Schneider, Rasband, & Eliceiri, 2012).

### 2.4 Statistical analyses

All statistical analyses were performed in RStudio version 0.99.467 (RStudio Team, 2015) and R version 3.3.3 (R Core Team, 2015) using the lme4 package (Bates, Maechler, Bolker, & Walker, 2015) to perform generalized linear mixed effects models (GLMMs), and package MASS (Venables & Ripley, 2002). For the analysis of individual traits, we employed GLMMs with appropriate data distributions including selection regime as fixed factor and individual line IDs and block as random effects to account for replicate line and block effects and if needed additionally used an observation level random factor to correct for overdispersion (Harrison, 2014, 2015). The respective data distributions used are given with the results. We note that by including selection line ID as a random effect in mixed models, these represent our level of replication for selection regimes. Throughout the result section, we chose to report the total number of observations as this indicates the number of animals used in each experiment. Model selection was performed based on *p* values using likelihood ratios of nested models compared to a χ^2^ table.

We conducted a principal component analysis (PCA) combining the above‐measured male traits to gain insight into how these traits contribute to variation between individual lines and the imposed selection regimes. Further, we determined Euclidean distances of each selection line to its selection regime‐specific center to test whether selection lines in the P selection regime diverged stronger from each other than selection lines in the M selection regime as sexual selection and sexual conflict are expected to lead P lines along independent evolutionary trajectories (Fricke, Andersson, & Arnqvist, 2010). For both analyses, selection line means for the different male traits were needed. As experiments were carried out in two blocks, we included the data from the Dahomey wild type that was measured in both blocks in the statistical model to estimate the block effect. To that end, we fitted generalized linear models (GLMs) with line ID and block as additive explanatory variables with appropriate data distributions (Table 2). We then used predicted values for block I for all selection lines as line means to make values for block I and block II comparable. Although half of the lines were not measured in block I, the models could predict the values for these lines for block I based on the differences in the Dahomey wild‐type strain between the two blocks. For male body size (wing length), a different approach was taken as the Dahomey strain was not measured and could not be used to determine block effects. Here, wing length was first modeled against block, and model residuals were then taken to model line differences. Details on the different models can be found in Table 2. To capture as much between‐selection line variance as possible without including differences occurring just by chance, a *p* value cutoff of *p *=* *.1 was taken, excluding two of the ten estimated traits (female remating rate and female LRS). The remaining eight traits were analyzed using a PCA with scaled (to unit variance) and centered values as response variables. Additionally, Euclidean distances between selection lines and their selection regime‐specific centers were determined using *z*‐transformed values (mean = 0 and standard deviation = 1).

**Table 2:** Detailed information on GLMs used to predict individual selection line means for male reproductive traits used for PCA and measures of Euclidian distances. Traits with a *p* value > .1 were not included in the PCA and the measures of Euclidian distances

Trait measured (Experiment)	Abbr.	Data distribution	*n*	*df*	Test statistic	*p* value
Sperm defense (B)	P1	Binomial (with quasi ext.)	718	20	*F *=* *2.89	<.001
Paternity share in direct competition (C)	PS	Binomial (with quasi ext.)	591	20	*F *=* *1.56	.0568
Mating share in direct competition (C)	MS	Binomial	586	20	χ^2^ = 53.05	<.001
Mating latency (D)	ML	Gamma	1,043	20	*F *=* *3.79	<.001
Copulation duration (D)	CD	Gamma	1,036	20	*F *=* *3.72	<.001
Female 24 hrs fecundity (D)	FE	Negative binomial	492	20	χ^2^ = 29.36	.0808
Proportion of females remating (D)	–	Binomial	1,025	20	χ^2^ = 17.79	.6011
Female LRS (E)	–	Negative binomial	418	20	χ^2^ = 8.23	.9902
Female life span (E)	LS	Gamma	418	20	*F *=* *1.45	.0962
Wing length (F)	WL	Gaussian	398	19	*F *=* *8.10	<.001

Graphs were made using the gplots package (Warnes et al., 2016). Unless otherwise stated, we present means with standard errors calculated from raw data.

## 3 RESULTS

### 3.1 (A) Female mating frequency and population fitness within selection lines

We found that selection regime did not affect the number of matings per female in our selection lines (Poisson data distribution: $\chi_{1}^{2}$ = 0.03, *p *=* *.85, *n* = 851). Females in both regimes mated several times during the observation period. Interestingly, number of matings was instead significantly affected by the actual mating setting females were exposed to; that is, if females were held in groups or individually with one male ($\chi_{1}^{2}$ = 7.13, *p *=* *.008, *n* = 851; Table 3). Females held in pairs mated more often than females held in groups (monogamous setting: 1.99 ± 0.05 matings per female; polygamous setting: 1.72 ± 0.05).

**Table 3:** Phenotypic tests of male traits after manipulating the opportunity for sexual selection and sexual conflict for more than 50 generations in 20 replicate selection lines (*n* = 10 per treatment) using *D. melanogaster*. We present the traits measured, in which generation assays were performed and the response averaged over the ten monogamous respective ten polygamous lines. Data presented are means (±*SE*) with sample sizes in parentheses

Trait measured (Experiment)	Generation	Monogamous				Polygamous			
(A) Population fitness (no. of adult offspring)	51/52	Monogamous setting 62.7 ± 1.6 (195)		Polygamous setting 61.2 ± 1.5 (159)		Monogamous setting 64.7 ± 1.3 (204)		Polygamous setting 62.7 ± 1.6 (195)	
	79	Monogamous setting 56.9 ± 1.7 (207)		Polygamous setting 51.4 ± 1.5 (210)		Monogamous setting 59.3 ± 1.6 (206)		Polygamous setting 57.6 ± 1.5 (203)	
(B) Sperm defense (P1)	60/61	19.8% ± 2.2 (317)				21.1% ± 2.3 (324)			
(C) Paternity share in direct competition	72/74	34.9% ± 2.9 (264)				43.6% ± 3.1 (250)			
(C) Mating share in direct competition	72/74	39.0% ± 3.0 (264)				55.3% ± 3.1 (250)			
(D) Proportion of females remating	54/55	24 hrs 19% ± 4 (125)	48 hrs 48% ± 5 (118)	72 hrs 70% ± 4 (115)	96 hrs 91% ± 3 (100)	24 hrs 15% ± 3 (126)	48 hrs 44% ± 5 (121)	72 hrs 77% ± 4 (116)	96 hrs 86% ± 4 (104)
(D) Copulation duration (minutes)	54/55	18.7 ± 0.2 (465)				17.8 ± 0.2 (472)			
(D) Mating latency (minutes)	54/55	45.9 ± 2.2 (469)				29.9 ± 1.8 (474)			
(D) Female 24 hrs fecundity	54/55	45.5 ± 1.0 (230)				43.5 ± 1.1 (233)			
(E) Female LRS (no. of adult offspring)	62/63	307 ± 12 (189)				292 ± 12 (190)			
(E) Female life span (days)	62/63	20.1 ± 0.5 (189)				19.7 ± 0.5 (190)			
(F) Male wing length (pixels)	85	4,356.63 ± 8.17 (199)				4,406.38 ± 8.27 (199)			

Population fitness measured as the number of adult offspring produced per female was not significantly affected by selection regime, actual mating setting or their interaction after (1) 51/52 generations (negative binomial data distribution: selection regime: $\chi_{1}^{2}$ = 0.71, *p *=* *.40, *n* = 753; mating setting: $\chi_{1}^{2}$ = 0.66, *p *=* *.42; interaction: $\chi_{1}^{2}$ = 0.01, *p *=* *.91; Table 3) or (2) 79 generations (negative binomial data distribution: selection regime: $\chi_{1}^{2}$ = 1.44, *p *=* *.23, *n* = 826, mating setting: $\chi_{1}^{2}$ = 2.93, *p *=* *.09, interaction: $\chi_{1}^{2}$ = 0.95, *p *=* *.33; Table 3) of selection.

### 3.2 (B) Sperm competition

The proportion of offspring sired by the selected male when first to mate with a tester female of two (sperm defense, P1) was not significantly affected by selection regime (binomial data distribution: $\chi_{1}^{2}$ = 0.52, *p *=* *.47, *n* = 641; Table 3).

### 3.3 (C) Direct competition

**Figure 1:**
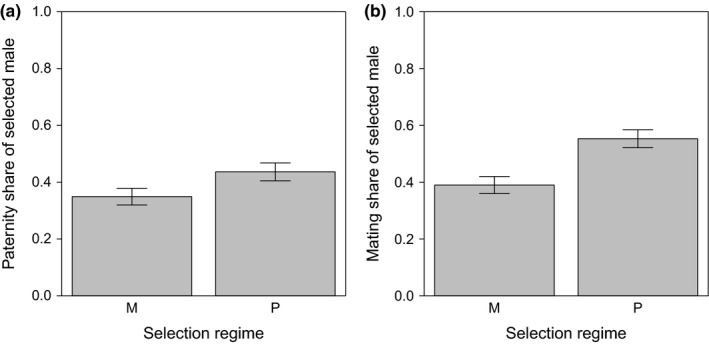
Selected male competitiveness when in direct competition with a *Sb* male for one tester female. Mean proportion (±*SE*) of (a) offspring sired and (b) matings gained by males maintained either under monogamy (M) or polygamy (P) for 72/74 generations prior to testing. Tester females were from an inbred line which was generated from a single Dahomey wild‐type pair and had undergone ten generations of full‐sib matings

When in direct competition with a competitor male for one female over several days, P males gained a significantly higher paternity share compared to M males (binomial data distribution: $\chi_{1}^{2}$ = 6.03, *p *=* *.014, *n* = 514; Figure 1a). P males were also more successful in gaining a mating as they achieved a higher proportion of matings compared to M males (binomial data distribution: $\chi_{1}^{2}$ = 16.61, *p *<* *.001, *n* = 514; Figure 1b). The higher mating share of P males significantly explained the higher paternity share (mating share included as a covariate: $\chi_{1}^{2}$ = 54.47, *p *<* *.001, *n* = 514), while the explanatory power of selection regime disappeared ($\chi_{1}^{2}$ = 1.41, *p *=* *.24, *n* = 514).

### 3.4 (D) Selected males’ ability to prevent female remating

**Figure 2:**
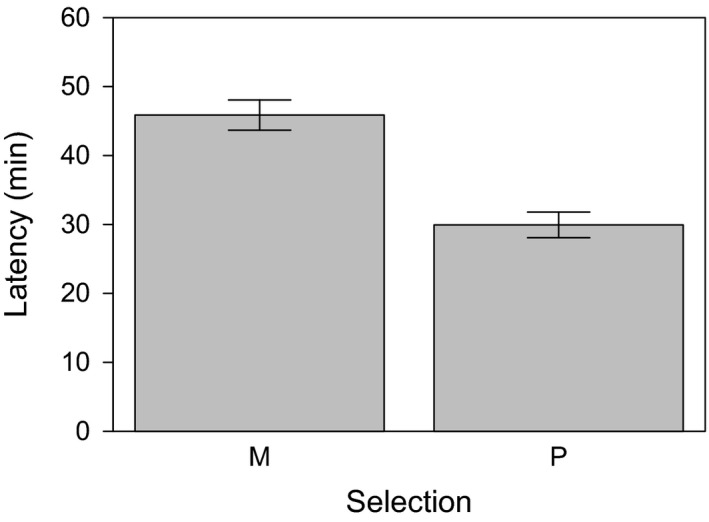
Mean (±*SE*) latency time from first introduction until mating of selected males with virgin tester females. Selected males were either maintained under monogamy (M) or polygamy (P) for 54/55 generations prior to testing. Tester females were from an inbred line which was generated from a single Dahomey wild‐type pair and had undergone ten generations of full‐sib matings

P males were faster in starting a mating with a virgin tester female than M males (gamma data distribution with log‐link function: $\chi_{1}^{2}$ = 13.96, *p *<* *.001, *n* = 943; Figure 2), but there was no effect of selection regime on copulation duration (gamma data distribution with log‐link function: $\chi_{1}^{2}$ = 1.26, *p *=* *.26, *n* = 937; Table 3). The proportion of females that remated increased steadily from 17 ± 2.4% at 24 hrs to 88 ± 2.3% at 96 hrs. While females were more likely to mate a second time as more time had elapsed since their first mating (binomial data distribution: $\chi_{1}^{2}$ = 301.24, *p *<* *.001, *n* = 925), M and P selection line males did not differ in their ability to repress female willingness to remate ($\chi_{1}^{2}$ = 0.48, *p *=* *.49, *n* = 925; Table 3) nor did the interaction between selection regime and time span between the two mating opportunities significantly affect remating proportions ($\chi_{1}^{2}$ = 3.39, *p *=* *.33, *n* = 925). Neither did male selection regime affect the number of eggs laid after a single mating (negative binomial data distribution: $\chi_{1}^{2}$ = 0.90, *p *=* *.34, *n* = 463; Table 3).

The analyses of the latency and copulation duration data from the sperm competition experiment (B) yielded qualitatively similar results as presented here (gamma data distribution with log‐link function: latency: $\chi_{1}^{2}$ = 5.85, *p *=* *.016, *n* = 1,089; copulation duration: $\chi_{1}^{2}$ = 2.00, *p *=* *.16, *n* = 1,084), thus indicating that this result is robust.

### 3.5 (E) Induction of female harm by selected males

Male selection regime did not affect tester females’ estimate of LRS (negative binomial data distribution: $\chi_{1}^{2}$ = 0.40, *p *=* *.53, *n* = 379) and life span (gamma data distribution with log‐link function: $\chi_{1}^{2}$ = 0.11, *p *=* *.74, *n* = 379) when being continuously exposed to males (Table 3).

### 3.6 (F) Male body size

Selection regime had a marginally nonsignificant effect on male wing length (Gaussian data distribution: $\chi_{1}^{2}$ = 3.40, *p *=* *.065, *n* = 398) which was used as a proxy for male body size. P males had a tendency to be larger than M males (Table 3).

### 3.7 Principal component analysis

**Figure 3:**
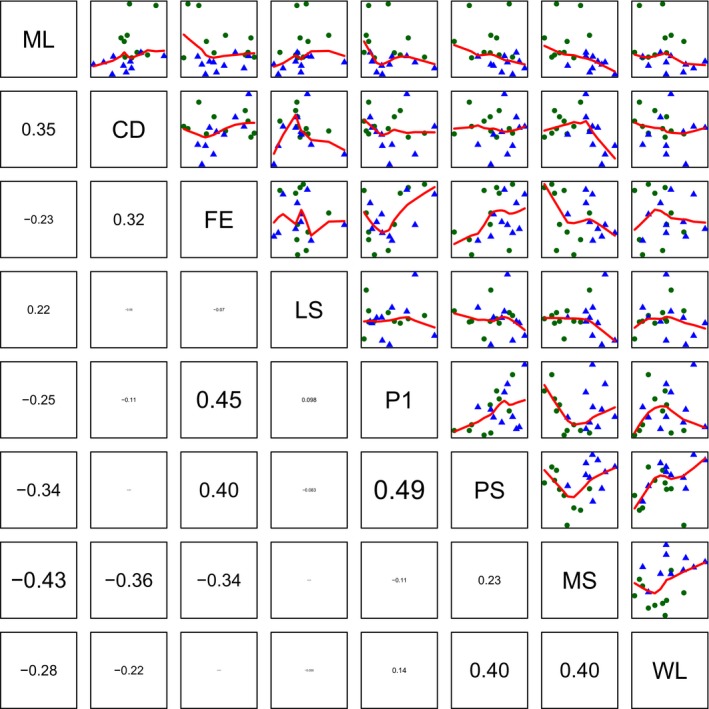
Pair plot of male reproductive traits with Pearson's correlation coefficients in the panels in the lower diagonal and scatterplots with smoothing lines in the panels in the upper diagonal. Font size of Pearson's correlation coefficients is proportional to the absolute value. Green circles represent individual monogamous selection lines, and blue triangles represent individual polygamous selection lines (CD, copulation duration; FE, female 24 hrs fecundity after a single mating; LS, female life span; ML, latency until mating; MS, mating share in direct competition; PS, paternity share in direct competition; P1, sperm defense; WL, wing length)

**Figure 4:**
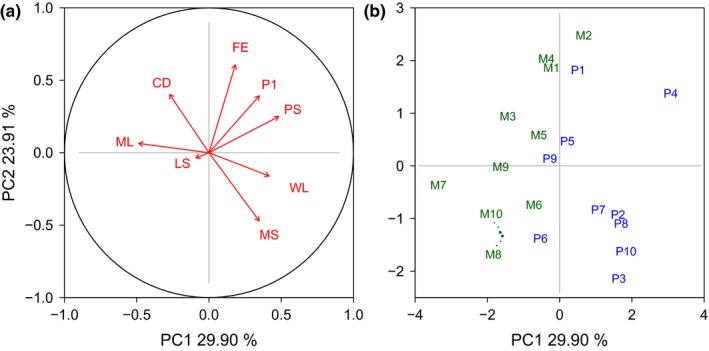
Principal component analysis of reproductive traits measured for selected males. (a) Loadings of male reproductive traits along the first two principal components (CD, copulation duration; FE, female 24 hrs fecundity; LS, female life span; ML, mating latency; MS, mating share in direct competition; PS, paternity share in direct competition; P1, sperm defense; WL, wing length). (b) Projection of all 20 selection lines on the first two principal components (M: monogamy treatment, P: polygamy treatment; numbers indicate replicate lines)

**Figure 5:**
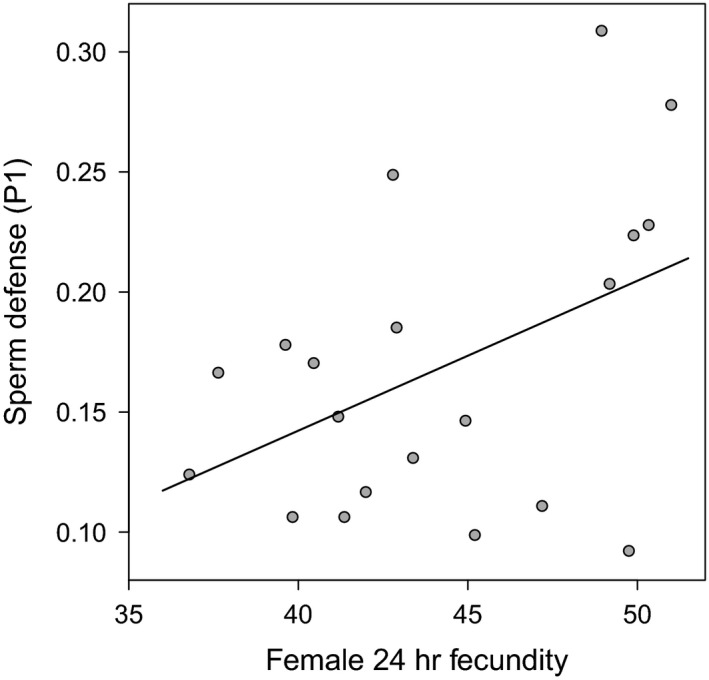
Correlation of tester female 24 hrs fecundity and selected male paternity share in a double mating experiment when first to mate with a tester female (sperm defense, P1). Points represent individual selection line means predicted from GLMs. Best‐fit line was obtained through least square method. Selected males were maintained either under monogamy or polygamy for 54/55 generations (fecundity) 60/61 (P1), respectively, prior to testing. Tester females were from an inbred line which was generated from a single Dahomey wild‐type pair and had undergone ten generations of full‐sib matings

Several of the measured male traits showed considerable correlation (for a pairs plot including Pearson's correlation coefficients, see Figure 3); therefore, it is not surprising that the first two principal components (PCs) already contained about 55% of the total variation between individual selection lines (Table 4). Selection lines from the two selection regimes were clearly separated by PC1 (Figure 4b) showing that some of the measured traits responded to the imposed selection regimes. Scores for PC1 were significantly different between selection lines from the P and M selection regime (GLM, Gaussian data distribution, *F*_1,19_ = 19.36, *p *<* *.001, *n* = 20), while no difference could be observed in the other PCs. The fact that selection lines from the different selection regimes were separated by PC1 indicates that a large proportion of the variance in the measured traits between lines were created by the imposed selection regimes. The traits latency to mating, wing length, and paternity share gained in the direct competition assay showed the strongest correlation with PC1 (Table 4) confirming the results of the individual trait analyses that identified precopulatory traits to respond strongest to selection regime. Wing length and latency to mating pointed in opposite directions (Figure 4a), indicating that large males tended to have shorter mating latencies, and (probably as a result) these males tended to have a higher paternity share in direct competition. PC2 showed the strongest correlation with female fecundity, mating share as measured in the direct competition assay, copulation duration, and P1 success, and was thus a mixture of pre‐ and postcopulatory traits. Interestingly, the number of eggs laid by tester females mated to selected males (female 24 hrs fecundity) and P1 pointed in similar directions, indicating a correlation of these two traits. This was tested using a Pearson's product–moment correlation coefficient. Lines showing high 24 hrs fecundity also show high P1 values (ρ = 0.45, *t*_18_ = 2.12, *p *=* *.049, *n* = 20), although there was considerable variation remaining unexplained (Figure 5). Surprisingly, female life span did not strongly contribute to PC1 and PC2 but dominated PC3, indicating little correlation between male‐induced harm and other traits measured in this study.

**Table 4:** PCA results. (A): Loadings of each measured male reproductive trait on the eight principal components. (B): Variance contained by the individual principal components

Trait measured	PC1	PC2	PC3	PC4	PC5	PC6	PC7	PC8
*(A) Loadings*								
Mating latency (ML)	−0.49	0.06	−0.23	0.4	−0.44	0.29	0.16	0.49
Copulation duration (CD)	−0.27	0.4	0.25	0.56	0.38	0.06	−0.45	−0.21
Female 24 hrs fecundity (FE)	0.18	0.61	0.07	−0.07	0.26	−0.36	0.32	0.54
Female life span (LS)	−0.09	−0.04	−0.87	0.14	0.38	−0.2	0.09	−0.16
Sperm defense (P1)	0.35	0.39	−0.36	−0.24	−0.3	0.23	−0.62	0.12
Paternity share in direct competition (PS)	0.48	0.25	−0.04	0.31	−0.03	0.52	0.48	−0.32
Mating share in direct competition (MS)	0.35	−0.47	0.02	0.21	0.44	0.31	−0.19	0.53
Wing length (WL)	0.42	−0.16	0	0.56	−0.4	−0.57	−0.1	−0.01
*(B) Variance*								
Proportion of variance contained	0.3	0.24	0.14	0.11	0.08	0.06	0.04	0.03
Cumulative proportion	0.3	0.54	0.68	0.79	0.87	0.93	0.97	1

### 3.8 Euclidean distances

Euclidian distances were not significantly different between the two selection regimes (GLM, Gaussian data distribution, *F*_1,19_ = 0.001, *p *=* *.98, *n* = 20), demonstrating equal divergence of lines under monogamous and polygamous selection regimes from their respective selection regime centers.

## 4 DISCUSSION

By enforcing monogamy, we eliminated selection on intrasexual male competition and any potential for sexual conflict and found that our mating system manipulations significantly affected male competitiveness. When in direct competition with another male for access to a female and for fertilizations, males that evolved under enforced monogamy gained a significantly lower proportion of matings and sired a significantly lower proportion of offspring compared to males that evolved under polygamy. Surprisingly, when testing for differences in postcopulatory traits, we found no differences between M versus P males in sperm defense ability or ability to prevent females from remating. Instead, males from M lines gained fewer matings and needed longer to start a mating. We strongly expected postcopulatory competitive traits to diverge between the two selection regimes due to the relaxation (M regime) or prevalence (P regime) of postcopulatory sexual selection. Intrasexual competition between males in the P regime should maintain or even enhance competitiveness of such traits, while, assuming that such traits are costly (Simmons, 2001), we expected males in the M regime to reduce expression of those traits under relaxed sexual selection. Surprisingly when testing individual traits, we found this to be true for precopulatory traits, but M males maintained their postcopulatory competitiveness despite not having encountered sperm competition for more than 50 generations. At the same time, M males also did not become less harmful to females.

As we measured a number of different male reproductive traits, we used a PCA approach to gain a comprehensive picture of the variance between our selection lines. PC1 accounts for 30% of the variance in measured traits between the 20 selection lines and significantly separated the two regimes. The difference in latency to mating strongly contributed to separating male reproductive phenotypes between the two selection regimes as revealed by the high loading this trait has on PC1 (Table 4). Mating latency was opposed by male wing length as a proxy for body size. It is well known that larger males are better in gaining a mating (e.g., Pitnick, 1991; Friberg & Arnqvist, 2003) and here also seem faster to do so. Both male body size and mating latency might have affected male mating share as measured in the direct competition assay (C). Mating latency is strictly speaking not a male trait as females need to accept males as mates and thereby control the start of mating (Spieth, 1974; Ritchie, Halsey, & Gleason, 1999). However, mating latency also reflects male traits such as attractiveness and courtship behavior (which we did not measure in this study) which both influence a female's decision to accept a mating. Our data show that M males were inferior in this respect and were accepted less quickly by females. When in direct competition with a competitor, the longer latency to mating put M males at a disadvantage, and they overall gained a lower proportion of matings which resulted in a reduced offspring share (Figure 1). Hence, this reduction in male precopulatory abilities directly reduced male reproductive success when in competition. We found significant variance between our selection lines for most of our traits, but for postcopulatory traits there was no distinct selection regime‐dependent response. Therefore, the pertinent question is why we found a selection regime‐specific signature for pre‐ but not postcopulatory traits in our selection lines.

Lack of divergence of postcopulatory traits might be explained by low heritabilities of traits determining sperm competition success (Bjork, Starmer, Higginson, Rhodes, & Pitnick, 2007; Morrow, Leijon, & Meerupati, 2008; Dobler & Reinhardt, 2016) or limits set by complex ejaculate × ejaculate and ejaculate × female interactions (Clark, 2002; Bjork et al., 2007). These factors make it difficult to directly select for increased sperm defense and offense performance in *D. melanogaster* (Bjork et al., 2007) despite evidence for high additive genetic variation for sperm competition success (Friberg, Lew, Byrne, & Rice, 2005; Bjork et al., 2007; Dobler & Reinhardt, 2016) and associated traits such as Sfps (Fiumera et al., 2005, 2007) and sperm traits (e.g., sperm length Miller & Pitnick, 2002).

Further, we only measured sperm defense and but not sperm offense in this study. Evidence points toward sperm defense and sperm offense not being genetically correlated (Clark, Aguade, Prout, Harshman, & Langley, 1995; Fricke, Martin, et al., 2010; Dobler & Reinhardt, 2016); hence, it is possible that sperm offense phenotypes evolved independently of sperm defense phenotypes here. However, data from a recent study found evolvability in sperm defense to be higher than in sperm offense (Dobler & Reinhardt, 2016). Taken together with previous unsuccessful attempts to select for sperm offense (Bjork et al., 2007), this suggest that sperm offense might be similarly unresponsive to our manipulation of postcopulatory selection.

Hence, the complex interactions between Sfps, sperm traits, and female reproductive tract morphology on sperm competition outcomes (Lüpold et al., 2012) can constrain the evolvability male postcopulatory traits (Bjork et al., 2007). Similarly, the evolution of such traits might be constrained by their positive effect on reproductive success independent of the intensity of postcopulatory selection. We found that tester females’ fecundity within 24 hrs after a single mating to a selected male and male sperm defense ability was correlated (Figure 5). Males in the M regime will equally benefit from eliciting high female fecundity, and therefore, we would not expect this trait to erode in M males, thereby possibly also maintaining male sperm defense ability.

While we found no difference in evolutionary change in postcopulatory traits in males, precopulatory traits clearly responded to our selection regime (Figure 2). This is in line with other studies showing that male courtship behavior evolved in response to different levels of male intrasexual competition with M males displaying courtship less frequently than P males in *D. melanogaster* (Holland & Rice, 1999) and *D. pseudoobscura* (Crudgington, Fellows, & Snook, 2010), while components of postcopulatory success did not diverge. As pointed out by Hosken and House (2011), gaining a mating or not might be the predominant factor determining male reproductive success, as a male who does not mate will not reproduce at all or partake in postcopulatory competition and therefore not experience postcopulatory selection. This is especially important in species with strong last male sperm precedence (Parker & Pizzari, 2010; Michalczyk et al., 2011; McNamara et al., 2016) such as *D. melanogaster*, as it might only then be beneficial to invest more in gaining a high number of matings as this increases the probability to be the last male and gain the majority of offspring. Indeed, a study partitioning variance in male reproductive success found that in *D. melanogaster,* mating success (precopulatory trait) and fertilization success (postcopulatory trait) contribute similarly to variation in male reproductive success. However, variation in fertilization success was largely due to mating order effects, and when adjusting for these, only 2% of male reproductive success is attributable to fertilization success and the larger fraction to mating success (Pischedda & Rice, 2012). Additionally, a study looking at “footprints” of intersexual coevolution by identifying male × female genotype interactions in cosmopolitan populations of *D. melanogaster* found evidence for such interactions in a precopulatory (mating speed) but not a postcopulatory (reproductive investment) trait (Pischedda, Stewart, & Little, 2012). Our results support the notion that mating success is an important determinant of male reproductive competitiveness in *D. melanogaster* and was responsive to our manipulation of selection pressures.

We tested two further hypotheses derived from sexual conflict theory. First, that males become more benign toward females when sexual conflict is removed (Holland & Rice, 1999) and second that populations diverge in reproductive traits due to perpetual sexually antagonistic coevolution (Rice, 1998; Gavrilets, 2000). With regard to the first idea, we here found no change in male‐induced harm toward females. Females continuously housed with M males did not have higher LRS or higher longevity compared to females continuously held with P males (Table 3). This might not be surprising considering that we also did not find divergence in male postcopulatory competitive traits that potentially contribute to eliciting the cost of mating in females as a side effect. Further, the results from the PCA show that male‐elicited changes to female life span contributed weakly to PC1 and PC2 but dominated PC3 indicating that it correlated little with any male reproductive trait measured here. We also found no evidence for the second hypothesis as M and P lines were equidistant from their selection regime‐specific centers.

Aside from biological reasons that might explain the lack of evolution in postcopulatory traits in our study, it is also possible that our experimental design affected the outcomes reported here. We might have imposed our selection regime for an insufficient amount of time to detect differences. However, with more than 50 generations of selection before testing phenotypes, we already selected for longer than most other studies that found significant effects (e.g., Holland & Rice, 1999; Wigby & Chapman, 2004; Nandy, Chakraborty, et al., 2013 and see Table 5). Importantly, we could show divergence in precopulatory traits according to selection regime, and hence, we conclude that our selection regime did enforce different selection pressures in our lines. Another concern often raised with respect to experimental evolution studies is the effective population size (Wigby & Chapman, 2004; Rice & Holland, 2005; Fricke & Arnqvist, 2007; Snook, Brüstle, & Slate, 2009). We calculated effective population sizes for our selection lines with census‐based estimators (Rice & Holland, 2005; Snook et al., 2009) and according to this method, the effective population sizes for both regimes should be >100 (M lines: *N*_e_ = 120, P lines: *N*_e_ ≈ 138). Therefore, *N*_e_ should be high enough for genetic drift and inbreeding to be of little concern (Snook et al., 2009) and for us to be able to detect a signature of our mating system manipulation.

**Table 5:** Overview of a literature search of different studies experimentally manipulating sexual selection and sexual conflict in a number of different invertebrate species. We focus on presenting results from tests of evolution in male reproductive traits with numbers in parentheses indicating the number of generations after which phenotypes were measured. Empty cells indicate that these traits were not measured. *N* refers to the total number of adults set up per replicated population

Organism	Manipulation of sexual conflict and sexual selection	Male competitive ability	Body size	Testes size	AG size	Sperm	Copulation duration	Courtship intensity	Mating latency	Male effect on tester/ancestral female harm	References
*Drosophila melanogaster*	Enforced monogamy (M); control polygamy (P) (1♀:3♂) *N* = 200	SC: no effect (81)	P > M (61)	P > M (61)		Numbers: P > M (81) length: mixed results (61&81)		P > M (45)		Fecundity: M > P Longevity: M > P (34)	(Holland & Rice, 1999; Pitnick et al., 2001)
	Enforced monogamy; control polygamy (5♀:5♂) *N* = 200	DC: P > M (88–114)	No effect (88–114)						No effect (88–114)		(Hollis & Kawecki, 2014; Hollis, Keller, & Kawecki, 2017)
	OSR bias (1:3, 1:1, 3:1) *N* = 450	SC: MB > FB (55–60)	No effect ( > 140)	No effect ( > 140)	No effect ( > 140)		MB > FB (55–60)	MB > FB (51–55)		Fecundity: FB > MB (45–47) Longevity: FB > MB (50)	(Chechi et al., 2017; Nandy, Chakraborty, et al., 2013; Nandy, Gupta, et al., 2013)
	OSR bias (3:1, 1:1, 1:3) *N* = 100		No effect (32)	No effect (32)	No effect (32)		No effect (60, 65, 67)	No effect (33)	No effect (60, 65, 67)	Longevity: no effect (33)	(Linklater, Wertheim, Wigby, & Chapman, 2007; Wigby & Chapman, 2004)
	Female OSR bias (1:1, 4:1, 10:1) *N* = 100, 156, 303			10:1 > 4:1 = 1:1 (28)	No effect (28)						(Reuter et al., 2008)
*Drosophila pseudoobscura*	Enforced monogamy, control polygamy (C: 1♀:3♂) and elevated polygamy (E: 1♀:6♂) *N* = 160			No effect (63–67)	E > M (71–78)	Numbers: no effect length: no effect (42/43)		E > M = C (91/92)		LRS: M > E (54/55)	(Crudgington et al., 2009, 2010)
*Scathophaga stercoraria*	Enforced monogamy, postcopulatory polygamy (1♀:3♂)	SC: P > M (10)	No effect (10)	P > M (10)		Length: no effect (10)	No effect (10)				(Hosken & Ward, 2001; Hosken et al., 2001)
*Tribolium castaneum*	OSR bias (9:1, 1:1, 1:6) *n* = 100/105	SC: with control females: no effect (30); with coevolved females: MB > FB (77) DC: MB > FB (22)				Length: MB > FB (77)	MB > FB (32)	MB > FB (32)	FB > MB (32)		(Godwin et al., 2017; Michalczyk et al., 2011)
*Callosobruchus maculatus*	Enforced monogamy, control polygamy (60♀:60♂) *N* = 120			P > M ( > 90)		Length: no effect ( > 90)					(Gay et al., 2009)
	OSR bias (1:2, 2:1) *N* = 120	SC: No effect (32)		No effect (32)	No effect (32)		No effect (32)				(McNamara et al., 2016)
*Onthophagus taurus*	Enforced monogamy, control polygamy (10♀:10♂) *N* = 120	SC: P > M when competing against each other (11/16)	No effect (6–20)	P > M (21)							(Simmons & García‐Gonzalez, 2008)
*Rhizoglyphus robini*	Enforced monogamy, control polygamy (5♀:5♂) *N* = 100	DC: P > M (37)	P > M (37)							Fecundity: P > M in M females, no effect in P females Longevity: M > P in M females (37)	(Tilszer, Antoszczyk, Salek, Zajac, & Radwan, 2006)

SC, sperm competition; DC, direct competition; MB, male‐biased; FB, female‐biased.

To put our results into context and synthesize findings from other studies, we conducted a search using Web of Science and the following search terms: experimental evolution and sexual selection. We selected studies with a focus on experimental evolution manipulating sexual selection and sexual conflict, measuring effects on male reproductive phenotypes and female costs of mating. Most of the relevant studies were performed in invertebrates, and we focus on those studies here. As a notable exception, there is a set of studies performed in mice to investigate changes in sperm, testes, and genital morphology in response to long‐term manipulation of sexual selection (Firman, Cheam, & Simmons, 2011; Firman et al., 2015; Firman & Simmons, 2014). For invertebrates, we found 19 publications from altogether twelve different experimental evolution studies (see Table 5) that fitted our criteria, and although we took every measure to be thorough in our literature search, we might have unintentionally overlooked relevant studies. Most of these studies were conducted with *Drosophila,* but also several coleopteran species were used. Selection pressures were altered by either enforcing monogamy or allowing polygamy or by manipulating the operational sex ratio (OSR). We found no indication that mode of manipulation had an impact on trait evolution.

Across all studies, measures of male precopulatory traits include mating latency and copulation duration as well as courtship intensity; for postcopulatory traits sperm competition, sperm morphology and reproductive tissue size were measured. When comparing results for male‐induced harm, four of five studies found males evolved under reduced levels of competition to induce fewer costs of mating in females indicating that the opportunity for sexual conflict was successfully manipulated, and only two including ours found no evidence of changes in male‐induced harm to females. In terms of postcopulatory traits, only seven studies compared success in sperm competition. Of these, four found an effect in the predicted direction with males evolved with a history of no sperm competition being inferior, while the other three studies and our own found no effect (Table 5). While half of the studies demonstrated the expected response in testes size, change in AG size was found only once. Sperm length was measured in five independent studies, and only one found sperm length to diverge in response to manipulation of sexual selection and conflict (Godwin et al., 2017; Table 5). Hence, adaptive changes in the reproductive tissue respective sperm morphology linked with success in sperm competition are elusive and have only been demonstrated in a few cases. Two studies (Michalczyk et al., 2011; Hollis & Kawecki, 2014) measured male competitive success when in direct competition for several days and not just in controlled double mating experiments and like us both found polygamous males to fare better. However, only one study (Michalczyk et al., 2011) additionally investigated individual male pre‐ and postcopulatory traits. They also found no differences in male sperm competitiveness but instead differences in precopulatory traits potentially explaining the advantage polygamous male held in direct competition in accordance with our results here. Similarly, of the seven studies that tested for differences in precopulatory traits, five highlighted a significant increase in courtship intensity in males evolved under intrasexual competition. For copulation duration and mating latency, the results are mixed though (half found evidence for longer copulation and shorter latencies after evolution under intense male–male competition, while the other half did not find any changes in these traits). Thus, while mixed and contradictory results occur both across and within species, our synthesis reveals that 50% of studies showed no difference in postcopulatory traits such as sperm competition or testes size due to selection regime, while 70% established polygamous males to court more. Hence, it seems that in general, precopulatory traits tend to be more responsive to manipulations of sexual selection. Our synthesis did not reveal any patterns that might explain the observed contradictory results. While experimental evolution assays are a powerful tool to manipulate specific aspects of a system and observe evolution in real time, there are also caveats that may have been underestimated in the past. Edward et al. (2010) summarized these caveats as (1) sexual conflict not being manipulated, (2) inadvertent selection, (3) differences in effective population sizes, (4) laboratory conditions masking differences, (5) differential gene × environment interactions, and (6) level of replication. With the exception of effective population size, the other caveats have not been addressed in detail in most studies. This might be due to the fact that often they are difficult to determine, such as inadvertent selection. Discussion of these caveats in relation to previous studies exceeds the scope of this manuscript; however, the accumulating evidence of unexpected outcomes calls for an in‐depth review of the strengths but also the limits of experimental evolution studies manipulating sexual conflict.

In summary, while we found evolutionary responses in male reproductive traits to our manipulation of sexual selection and sexual conflict regimes, we observed divergence in male precopulatory traits and not as expected in postcopulatory traits. Males who evolved in the absence of intrasexual competition were slower in gaining a mating, and when put in direct competition, this resulted in reduced reproductive success. Collectively, our data together with results from other studies indicate that we need to take into account a broad spectrum of traits to fully capture the evolutionary responses in male reproductive success to altered sexual selection pressures.

## CONFLICT OF INTEREST

None declared.

## AUTHOR CONTRIBUTIONS

KUW and CF designed the study. KUW carried out experiments. KUW and MK analyzed the data. KUW, MK, and CF wrote the manuscript.
